# Editorial: Biomarker Detection Algorithms and Tools for Medical Imaging or Omics Data

**DOI:** 10.3389/fgene.2022.919390

**Published:** 2022-05-25

**Authors:** William C. Cho, Fengfeng Zhou, Jie Li, Lin Hua, Feng Liu

**Affiliations:** ^1^ Department of Clinical Oncology, Queen Elizabeth Hospital, Hong Kong SAR, China; ^2^ College of Computer Science and Technology, Jilin University, Changchun, China; ^3^ School of Computer Science and Technology, Harbin Institute of Technology, Harbin, China; ^4^ Institute of Biomedical Engineering, Capital Medical University, Beijing, China; ^5^ School of Mechanic and Power Engineering, Wuhan University, Wuhan, China

**Keywords:** biomarker, detection algorithm, medical imaging, omics, artificial intelligence

Biomarkers are characteristics that can be objectively detected and assessed and can be used as indicators of normal biological processes, pathological processes, or pharmacological responses to therapeutic interventions. In the clinical aspect, biomarkers play a crucial role in the early diagnosis and classification of diseases, the judgment of disease degree, test of treatment effect, and prevention of disease. Therefore, some biomarker detection algorithms based on statistical models and artificial intelligence models have been constructed. However, there are still many issues in the existing algorithms, especially the high-performance algorithms to detect biomarkers of complex disease, such as cancer.

Traditional biomarker detection methods based on manual experimental methods are compl ex, inefficient, and costly. With the wide application of sequencing technology and digital imaging technology in biomarker detection, digital multi-omics data and medical images can be obtained rapidly and massively, providing the possibility for systematically detecting the characterization of disease, pathological causes, and data basis for algorithm-based automated biomarker detection. It is particularly important to combine multi-omics data with medical imaging, design algorithms that can efficiently identify biomarkers, discover more valuable biomarkers, and through the systematic combination of these new technologies and traditional biotechnology systems, ultimately provide a research basis for researchers, and doctors. However, how the construction of novel biomarker detection algorithms and identification of high-performance and robust biomarkers are still challenging problems.

In order to further promote the development of biomarker detection algorithms and develop more innovative algorithms, we proposed this Research Topic, which provided a platform for collecting recent discoveries in new feature extraction and feature selection algorithms for machine learning and deep learning models based on medical imaging and/or omics (genome, transcriptome, epigenome, proteome, and metabolome) data.

In structural biology and computer science, the image processing step is to traditionally cluster 2D cryo-electron microscopy (cryo-EM) images according to projection angle. Lei and Yang designed a new model, cascade of denoising autoencoders (CDAE), which was an efficient cryo-EM image denoising model. The model consisted of stacked deep neural network blocks that progressively reduced noise. When comparing state-of-the-art image denoising methods with significantly enhanced clustering results, they achieved a very competitive peak signal-to-noise ratio. Furthermore, the quantification and visualization of CDAE showed good noise reduction performance in clustered single-particle cryo-EM images.

Conventional computed tomography (CT) is an important imaging technique for establishing disease diagnosis. Huang and Lu provided a case report using CT findings and histopathological features of primary liver carcinosarcoma (PLC). CT scans and three-stage enhanced scans were performed on the patients. Pathological features were analyzed. They concluded that the CT features observed in this study were very beneficial for the diagnosis of PLCs.

In recent years, exploring the diagnostic value of CT imaging and radiomics features in diseases has become a hotspot (Feng et al., 2022). For the classification of lung adenocarcinomas presenting as ground-glass nodules (GGNs) on CT, Zheng et al. studied 312 GGNs. Univariate and multivariate logistic regression was used to establish clinical models, minimum redundancy maximum relevance, least absolute shrinkage, and selection operator (LASSO) algorithms were used to select radiomics features, and construct radiomics models. A combined nomogram was developed based on the combined model and evaluated using its calibration curves and concordance indices. They found that the area under the curve (AUC) value was higher in both models compared to the individual clinical or radiomic models. They claimed that the nomogram served as a non-invasive and accurate predictive tool to help judge the aggressiveness of GGN before surgery and to help clinicians develop personalized treatment strategies.

It is well known that ultrasonography is an important step in ultrasound-guided diagnosis and treatment, but it is difficult to develop an ideal segmentation method due to strong imaging artifacts. Wu et al. proposed a novel boundary-guided multi-scale network to improve the performance of breast lesion segmentation in ultrasound images based on a feature pyramid network (FPN). First, they developed a boundary-guided feature enhancement module to enhance the feature maps of each FPN layer by learning the boundary maps of breast lesion regions. They then devised a multi-scale scheme to exploit information from different image scales to deal with ultrasound artifacts. The segmentation results were then generated by fusing the fine and coarse segmentation maps to accurately segment the breast lesion area from the ultrasound image and effectively remove the false detections due to boundary feature enhancement and multi-scale image information. Finally, they found that their results outperformed state-of-the-art methods.

Chronic rhinosinusitis with nasal polyps (CRSwNP) is a complex multifactorial disease with significant public health concerns, but its pathogenesis is still unclear. Noncoding RNAs have been reported to be promising biomarkers for various diseases. Among them, circular RNAs (circRNAs) are associated with inflammatory diseases. Therefore, Yu et al. studied the expression of circRNAs and microRNAs (miRNAs) in the CRSwNP group and control group. The biological functions of predicted abnormally expressed circRNAs and miRNAs were verified by qRT-PCR using Gene Ontology enrichment analysis and Kyoto Encyclopedia of Genes and Genomes pathway analysis. Differentially expressed circRNAs and miRNAs between CRSwNP and controls were found. Among them, the altered expressions of hsa-circ-0031593 and hsa-miR-145-5p are the strongest evidence for involvement in the occurrence and development of CRSwNP, as their AUCs were higher than other molecules tested in this study.

In diabetic patients with and without ischemic stroke, Abdelaleem et al. found high expression levels of LINC00657 and miR-9 in serum and significantly lower serum miR-106a in the diabetic patients without stroke compared to the control participants. They claimed that serum noncoding RNAs (TUG1, LINC00657, miR-9, and miR-106a) might serve as potential novel biomarkers for stroke in diabetes. Their research may reveal new therapeutic targets for treating diabetic patients with stroke.

Multi-omics data are often measured to enrich the understanding of the biological mechanisms of certain phenotypes. However, due to the complex relationships and high dimensionality of multi-omics data, it is difficult to relate omics features to certain biological features of interest. Below are some diseases that use multi-omics data/methods for biomarker discovery.

Hepatocellular carcinoma (HCC), the third leading cause of cancer-related death worldwide, is a heterogeneous tumor with a complex tumor microenvironment (TME). TME refers to the microenvironment formed by immune cells and their products in tumor tissues (Fu et al., 2019). Bai et al. constructed a novel risk scoring model with prognostic value to elucidate the tumor immune microenvironment of HCC. ESTIMATE algorithm, single-sample gene set enrichment analysis (GSEA), and CIBERSORT analysis were used to reveal the characteristics of the HCC tumor immune microenvironment. After multiple analyses, four glycolysis-related long noncoding RNAs (lncRNAs) were obtained. The risk scores constructed with the four lncRNAs were found to be significantly associated with the prognosis of the patients. Besides, the risk scores were significantly correlated with immune scores, immune-related features, infiltrating immune cells (such as B cells), and key immune checkpoint blockade (ICB) molecules (such as CTLA4). Furthermore, they showed that MIR4435-2HG had a significant effect on the overall survival of the samples and was strongly associated with ICB treatment in HCC patients.

On the other hand, increasing evidence suggests that the abnormal expression of autophagy-related genes (ARGs) plays an important role in the occurrence and development of HCC. Luo et al. studied the ARGs in HCC. They constructed ARG pairs using ARGs extracted from the Human Autophagy Database and Molecular Signatures Database. They then developed a prognostic model based on ARG pairs, using LASSO Cox regression to assess the prognosis of patients after hepatectomy. Finally, they combined the signatures with independent prognostic factors to construct a nomogram. Based on ARG pair signatures, they could classify patients into high- or low-risk groups. Survival analysis and receiver operating characteristic (ROC) curve analysis verified the validity of the signature (AUC: 0.786–0.828). This model has a more accurate predictive effect than most HCC prognostic models. Their study provides evidence for the importance of autophagy in the occurrence and development of HCC, as well as a potential biomarker for targeted therapy.

For the poor prognosis of HCC, the development of prognostic prediction models is of great significance. Zhang et al. have identified seven gene signatures associated with pyroptosis (BAK1, CHMP4B, GSDMC, NLRP6, NOD2, PLCG1, and SCAF11) to predict the prognosis of HCC patients. They constructed a novel LASSO Cox regression pyroptosis-related gene signature that could predict the prognosis of HCC patients. GSEA analysis further revealed novel signature-related mechanisms of immune responses in high-risk populations. Furthermore, they found that the expression of immune checkpoints was enhanced in the high-risk group, while m6A-related modifications were differentially expressed between the low- and high-risk groups.

In addition to autophagy and pyroptosis, recent studies have identified ferroptosis as a programmed cell death involved in regulating tumor biological behavior. Song et al. investigated the association between ferroptosis-related gene (FRG) expression profile and prognosis in esophageal squamous cell carcinoma (ESCC) patients based on The Cancer Genome Atlas and Gene Expression Omnibus (GEO). They developed a novel signature of FRGs, including ALOX12, ALOX12B, ANGPTL7, DRD4, MAPK9, SLC38A1, and ZNF419. A prognostic nomogram was then constructed combining clinical factors and risk scores. Their study demonstrates that ferroptosis-related features are a factor independently predicting ESCC risk and their prognostic risk models can predict ESCC prognosis.

Breast cancer subtypes are well-defined at the molecular level but difficult to classify using gene expression data. Jung et al. proposed a multi-omics analysis method, called multi-omics non-negative tensor decomposition for integrative analysis (MONTI), which aimed to select multi-omics features that could represent trait-specific features. They formed a three-dimensional tensor from the multi-omics data. They found that MONTI could well explain certain clinical attributes using multi-omics data. Furthermore, MONTI could detect subtype-specific genomes that were strongly regulated by certain omics, from which correlations between omics types could be inferred.

Various technological revolutions have occurred in recent years. Molecular assays based on transcriptome data are developing rapidly. Clinically, distinguishing benign from malignant thyroid nodules is challenging. Yang and Gong combined five independent transcriptomic studies to discover genetic signatures between benign and malignant thyroid nodules. Hundreds of differentially expressed genes were discovered by feature selection methods and weighted gene co-expression network analysis was performed to identify the modules of highly co-expressed genes. Ultimately, they identified four key genes (ST3GAL5, NRCAM, MT1F, and PROS1) involved in the pathogenesis of malignant thyroid.

Single-cell RNA sequencing (scRNA-seq) is emerging as one of the most powerful tools for uncovering disease complexity. scRNA-seq performs high-throughput sequencing analysis of epigenetics, transcriptomes, and genomes at the single-cell level, with the advantages of high-throughput and high resolution. The revelation of new cell subsets can focus disease initiation and progression on specific biological activities of specific cells. Regarding the complexity of the retina, Ying et al. reviewed the novel retinal cell subtypes and some specific gene markers discovered by scRNA-seq. Since the batch effects in scRNA-seq data are known to remain a hindrance when integrating disparate datasets, Zou et al. proposed a new deep learning-based method, deep mutual nearest neighbor (deepMNN), to correct for batch effects in scRNA-seq data. They searched for MNN pairs across different batches in a principal component analysis subspace. A batch correction network was then constructed by stacking the two residual blocks and further applied to remove batch effects. They demonstrated that deepMNN achieved better or comparable performance in qualitative analysis using uniform manifold approximation and projection plots and quantitative metrics (such as batch and cell entropy). Furthermore, deepMNN ran much faster than other methods for large-scale datasets. With these properties, deepMNN may be well suited for large-scale single-cell gene expression data analysis.

Absorption contrast between the terahertz (THz) frequency range of adipose and cancerous tissue allows the diagnosis of cancer by THz imaging. Even without external comparisons, Chen et al. have successfully demonstrated the ability of THz imaging to measure the volume of small breast cancers in a subcutaneous xenograft mouse model. They estimated the volumetric detection limit of a fiber-based THz scanning imaging system using a highly sensitive cryogenically operated Schottky diode detector to be less than 1 mm^3^, thus showing the potential application of this technique in early cancer diagnosis.

Pulmonary hypertension (PH) affects the normal function of human pulmonary arteries. Peripheral blood mononuclear cells are an ideal source for minimally invasive disease diagnosis. Liu et al. proposed an ensemble feature selection algorithm (EnRank) to integrate the ranking information of popular feature selection algorithms, including T-test, chi-squared test, ridge regression, and LASSO. Using PH patient data, the biomarkers detected by EnRank provided useful information from these four feature selection algorithms and achieved very good predictive accuracy in predicting PH patients.

Epilepsy is a complex chronic neurological disorder that affects the health of approximately 70 million patients worldwide. About one-third of people with epilepsy develop drug resistance. Han et al. performed bioinformatic analysis to explore potential diagnostic markers and the mechanisms of drug-resistant epilepsy. Weighted correlation network analysis was applied to genes in epilepsy patients downloaded from the GEO database to identify key modules. Genes resistant to carbamazepine, phenytoin, and valproate were screened using LASSO regression and support vector machine (SVM) recursive feature elimination algorithms. Finally, ingenuity pathway analysis (IPA) was used for disease and functional pathway and network analysis. They found that the joint analysis yielded 17 resistance genes to construct a three-class classification SVM model. ROC analysis showed that the model could accurately predict patient resistance. Protein-protein interaction (PPI) revealed that six resistance genes (CD247, CTSW, IL2RB, MATK, NKG7, and PRF1) might play a central role in drug resistance in epilepsy patients. Finally, IPA revealed that resistance genes (PRKCH and S1PR5) were involved in CREB signaling in neurons.

PPI networks are critical for predicting essential proteins. The fusion of multiple biological information can reduce the impact of false data in PPI, but inevitably generates more noisy data. Zhang et al. proposed a new non-negative matrix tri-factorization (NMTF)-based model to predict essential proteins. A weighted PPI network was first built using the topological features of the network. The NMTF technique was then performed to reconstruct the optimized PPI network with more potential PPIs. A final ranking score for each protein was calculated using the PageRank algorithm, where the protein’s subcellular localization and homology information were used to calculate the initial score. This study demonstrates that introducing NMTF can effectively improve the condition of PPI network and reduce the impact of noise on predictions.

The sparse canonical correlation analysis (SCCA) model is a well-known tool for identifying meaningful biomarkers in imaging genetics. However, most SCCA models contain only diagnostic status information, which poses challenges in finding disease-specific biomarkers. To overcome this obstacle, Ke et al. proposed a multi-task sparse canonical correlation analysis and regression (MT-SCCAR) model to reveal disease-specific associations between single nucleotide polymorphisms and quantitative traits derived from multimodal neuroimaging data in the Alzheimer’s disease (AD) Neuroimaging Initiative cohort. MT-SCCAR used complementary information carried by multi-perspective cognitive scores and encouraged the population sparsity of genetic variation. This study used MT-SCCAR to identify major genetic risk factors for AD, including rs429358. They found some patterns of association between genetic variants and brain regions.

Deciphering the effects of epigenetic alterations on regulatory elements requires innovative computational approaches that can benefit from massive epigenomic datasets, such as roadmaps or blueprints. Wang et al. developed a software named Integrative Ranking of Epigenetic Network of Enhancers to enable quantitative analyses of differential epigenetic modifications through an integrated network-based approach. The additive effects of alterations on multiple regulatory elements of the gene were considered. Using this tool, the authors have successfully identified many known cancer genes from publicly available cancer epigenome datasets.

The omics dataset has high dimensionality, and the relationship between omics features is very complex. Yao et al. proposed a method based on integrated swarm intelligence to identify key biomarkers and effectively reduce the feature dimension. It was an end-to-end method that only relied on the rules of the algorithm itself, without presets such as the number of filtered features. Furthermore, this method achieved good classification accuracy without excessive consuming computational resources.

With the development of multi-omics algorithms and the application of artificial intelligence, the automatic identification and classification of biomarkers have made great progress and have been widely used in biomarker detection research (Marshall et al., 2022).
